# 同时性多原发肺癌的预后及生存相关因素研究

**DOI:** 10.3779/j.issn.1009-3419.2017.01.03

**Published:** 2017-01-20

**Authors:** 海法 郭, 锋 毛, 辉 张, 杨波 裘, 屠阳 申

**Affiliations:** 200039 上海，上海交通大学附属胸科医院/上海市肺部肿瘤临床医学中心 Shanghai Chest Hospital, Shanghai Jiaotong University, Department of Shanghai Lung Tumor Clinical Medical Center, Shanghai 200039, China

**Keywords:** 同时性多原发肺癌, 外科手术, 危险因素, 生存分析, Synchronous multiple primary lung cancer, Surgical operation, Risk factors, Survival analysis

## Abstract

**背景与目的:**

同时性多原发肺癌（synchronous multiple primary lung cancer, sMPLC）既往属少见疾病，近年发病率呈持续上升趋势，但缺乏对其大样本的研究报道。本研究对357例sMPLC的临床病理资料进行分析总结，籍以为临床诊断、治疗及预后提供理论依据。

**方法:**

参考Martini-Melamed诊断标准和国际肺癌研究协会（International Association for the Study of Lung Cancer, IASLC）第8版肿瘤-淋巴结-转移（tumor-node-metastasis, TNM）分期标准，对357例sMPLC的临床病理资料进行分析。

**结果:**

357例患者中，双原发病灶269例（75.35%），三原发病灶65例（18.21%），四原发病灶及以上者23例（6.44%），最多者为8个病灶。病灶好发于上叶，特别是右上叶（35.77%, 298/833），病理类型以腺癌为主（95.56%, 796/833），鳞癌次之（2.40%, 20/833），腺癌亚型中以腺泡样为主的比例较高（70.81%, 313/442），分期以Ib期及以下为主（68.35%, 244/357）。病理类型相同者发病率（92.72%, 331/357）远高于不同病理类型（7.28%, 26/357），其中腺癌-腺癌比例较高（99.40%, 329/331）。sMPLC的3年总生存率（overall survival, OS）为91.93%，5年总生存率为84.37%，多因素生存分析显示，有吸烟史（*P*=0.012）、最大病灶直径大于2 cm（*P*=0.027）、淋巴结转移（*P*=0.015）和胸膜受累（*P*＜0.001）为影响sMPLC预后生存的独立危险因素。

**结论:**

sMPLC好发于右上叶，以腺癌最常见，腺泡样为主的亚型多见。吸烟史、最大结节直径、淋巴结转移和胸膜侵犯是影响sMPLC预后生存的独立危险因素。早期发现和积极的手术可以使sMPLC患者获得较好的预后。

## Abstract (en)

**Background and objective:**

Synchronous multiple primary lung cancer (sMPLC) is a sparse disease in the past, but there has been a gradual increase in the morbidity of sMPLC recently. However, studies on large sample have never been undertaken. The purpose of this study is to investigate the diagnosis, treatment and prognosis of sMPLC through analyzing the clinical data, and provide supports for the management of sMPLC.

**Methods:**

According to Martini-Melamed criteria, 357 patients were diagnosed sMPLC. The pathological staging is on the basis of the 8^th^ edition tumor-node-metastasis (TNM) staging from International Association for the Study of Lung Cancer (IASLC).

**Results:**

There were 269 patients with double primary lung cancer, 65 patients with triple primary lung cancer and 23 patients with four or more primary lung cancer. Lesions (68.55%, 571/833) were frequently in upper lobe, especially the right upper lobe. Adenocarcinoma (95.56%, 796/833) was the mainly pathological type, followed by squamous cell carcinoma (2.40%, 20/833). The acinar predominant subtype was the main part (70.81%, 313/442) of the all adenocarcinoma specimens. Most of the lesions (68.35%, 244/357) were stage Ib or low. Among the initial lesion and the following lesions, patients who had the same pathological type (92.72%, 331/357) were more than the different (7.28%, 26/357), of which adenocarcinoma-adenocarcinoma occupied the major proportion (99.40%, 329/331). The 3-year overall survival (OS) and 5-year overall survival were respective 91.93% and 84.37%. Multivariate analysis found that smoking history (*P*=0.012), the diameter of the maximum lesion (*P*=0.027), lymph node metastasis (*P*=0.015) and pleural invasion (*P* < 0.001) were the independent risk factors for prognosis.

**Conclusion:**

Tumours in patients with sMPLC are more frequently in the right upper lobe. Adenocarcinoma was the mainly pathological type. Smoking history, the diameter of the maximum lesion, lymph node metastasis and pleural invasion were the independent risk factors for prognosis. Early diagnosis and active operation can obtain better prognosis.

随着临床诊断技术的不断进步，肺部多发结节的检出率逐年增高，多原发肺癌的诊断率亦呈上升趋势^[1]^。多原发肺癌（multiple primary lung cancer, MPLC）是指在同一患者肺内同时或先后发生两个或两个以上的原发性恶性肿瘤，若两个肺部恶性肿瘤的诊断间隔时间在6个月之内，则称为同时性多原发肺癌（synchronous multiple primary lung cancer, sMPLC）。同时性多原发肺癌占我院同期肺癌的1.41%（357/25, 339），由于sMPLC发病率相对较低，以往缺乏大样本量的报道，本研究总结357例sMPLC的临床病理特点和预后生存分析，为临床诊断、治疗及预后提供理论依据。

## 1 资料和方法

### 1.1 病例选择及一般资料

收集2010年1月-2015年12月在上海市胸科医院行手术治疗的sMPLC患者资料，临床资料和术后生存随访资料均完整者357例，占我院同期肺癌患者的1.41%（357/25, 339），发病率低于国外报道^[2-4]^。病例的选择标准参考Martini关于sMPLC的临床诊断标准^[5]^，同时结合临床工作实践，须符合下列标准之一：①同时发生的各个癌灶分别为不同的病理组织类型；②多个癌灶的病理组织类型相同，但符合下列条件：各癌灶起源于不同的原位癌；癌灶位于不同侧的肺、不同肺叶或不同肺段；无远处及纵隔淋巴结转移；影像学诊断倾向于多原发肺癌。各个病灶具有独特的病理形态特征为诊断sMPLC的要点。

根据上述诊断标准，筛选出临床资料完整的sMPLC患者357例。其中，男性109例（30.53%），女性248例（69.57%），男女比例0.44:1。年龄28岁-80岁，中位年龄60岁。吸烟者（≥400年支）61例（17.09%），不吸烟者296例（82.91%）。肿瘤最大长径0.4 cm-9 cm，中位数1.5 cm。根据病灶的影像学表现，将病灶分为计算机断层扫描（computed tomography, CT）纵隔窗不显影的肺部磨玻璃阴影（ground glass opacity, GGO）和CT纵隔窗显影的肺部磨玻璃结节（ground glass nodule, GGN）。病灶影像学形态表现，GGO-GGO组231例（64.71%），GGN-GGO组92例（25.77%），GGN-GGN组34例（9.52%）。肿瘤位于不同侧肺叶者97例（27.17%），同侧不同肺叶者128例（35.85%），同一肺叶者132例（36.98%）。病灶数目为2个者269例（75.35%），3个者65例（18.21%），≥4个者23例（6.44%），病灶最多者为8个。357例患者均行肺叶、肺段或楔形切除+纵隔淋巴结清扫或采样。

### 1.2 方法

所有病例经2位资深病理医生阅片审核，结合临床表现和影像学特征，按照Martini-Melamed标准确诊，排除肺内转移癌。总生存期（overall survival, OS）的计算从第一次手术治疗日期开始至死亡当天或者末次随访时间。采用电话术后随访，末次随访日期为2016年9月30日。分期按照肺癌肿瘤-淋巴结-转移（tumor-node-metastasis, TNM）分期（第8版）进行，对各个病灶单独分期，最终分期以最高分期为准。

### 1.3 统计学方法

采用SPSS 20.0软件对数据进行统计学分析，采用*Kaplan*-*Meier*法进行生存分析，并进行*Log*-*rank*检验，采用*Cox*模型做多因素回归分析。检验水准*α*=0.05。以*P*＜0.05为差异有统计学意义。

## 2 结果

### 2.1 术后情况

所有入组患者均接受手术治疗，无围手术期死亡。265例（74.23%）行同期手术，92例（25.77%）分期手术，两次手术间隔1个月-6个月，中位间隔2个月。术后接受辅助化疗者85例（23.81%）。截止随访日期，死亡25例（7.00%），3年总生存率为91.93%，5年总生存率为84.37%。对于病灶位于不同肺叶的病例，多采用叶切+局切（36.13%）或局切+局切（23.25%）的手术方式，病灶均位于同一肺叶的病例，则多采用叶切（30.81%）的手术方式。

357例sMPLC患者共有833个原发肿瘤（详见表 1），其中298个（35.77%）位于右上叶、158个（18.97%）位于右下叶、71个（8.52%）位于右中叶、202个（24.25%）位于左上叶和104个（12.48%）位于左下叶；就单个肺叶而言，肿瘤好发于右上叶（35.77%）；就整体而言，上叶肿瘤（68.55%）多于下叶（31.45%）。

**1:** 各病灶的临床特征 Clinical characteristics of the lesions

Clinical characteristics	Number (%)
Total	833 (100%)
Location	
Right upper lobe	298 (35.77%)
Right middle lobe	71 (8.52%)
Right lower lobe	158 (18.97%)
Lef tupper lobe	202 (24.25%)
Left lower lobe	104 (12.48%)
Pathological pattern	
Adenocarcinoma	796 (95.56%)
Squamous carcinoma	21 (2.52%)
Large cell carcinoma	9 (1.08%)
Pulmonary carcinoid	3 (0.36%)
Adenosquamous carcinoma	2 (0.24%)
Pleomorphic carcinoma	1 (0.12%)
Mucosa associated lymphoid tissue type (MALT)	1 (0.12%)
Imageological characteristics	
Ground glass nodule (GGN)	172 (20.65%)
Ground glass opacity (GGO)	661 (79.35%)

### 2.2 sMPLC病理学特征

病理类型组合以腺癌-腺癌最多（92.16%），腺癌-鳞癌次之（3.08%），其他组合占4.76%。其他组合包括鳞癌-大细胞癌5例，腺癌-大细胞癌4例，鳞癌-鳞癌、腺癌-类癌、与腺癌-腺鳞癌各2例，腺癌-肺淋巴瘤与腺癌-多形性癌各1例。各病例的T分期以各病灶最高T分期（第8版分期）为据，其中pTis 11例（3.08%），pT1a 85例（23.81%），pT1b 148例（41.45%），pT1c 73例（20.45%），pT2a 26例（7.28%），pT2b 8例（2.24%），pT3 4例（1.12%），pT4 2例（0.56%）。319例无纵隔淋巴结转移，38例出现纵隔淋巴结转移，其中，pN0 319例（89.36%），pN1 15例（4.20%），pN2 23例（6.44%）。胸膜侵犯者65例（18.21%），无胸膜侵犯者292例（81.79%）。

357例sMPLC患者共有833个原发肿瘤，其中，腺癌最多，占95.56%（796/833），鳞癌次之，占2.40%，余为其他各类型的肺部原发恶性肿瘤。在796个腺癌中，浸润性腺癌442个，微浸润腺癌191个，原位腺癌163个；影像学表现上，浸润性腺癌有68.55%的病灶表现为GGO形态，微浸润腺癌中97.91%表现为GGO，原位腺癌100%为GGO形态。在浸润性腺癌中，腺癌亚型以腺泡样为主，占70.81%（313/442），其次是乳头样，占26.24%，余为实体型和微乳头型，分别占1.58%和1.36%。

### 2.3 生存分析

根据表 2可知，单因素分析中有意义的临床因子分别为：①性别（男性，HR=5.090，95%CI：2.105-12.310，*P*＜0.001）；②年龄（＞60岁，HR=2.951，95%CI: 1.222-7.126，*P*=0.016）；③吸烟情况（吸烟，HR=5.023，95%CI: 2.238-11.273，*P*＜0.001）；④各肿瘤长径之和（＞3 cm, HR=8.419, 95%CI: 2.497-28.394, *P*=0.001）；⑤最大肿瘤的长径（＞2 cm, HR=9.269, 95%CI: 3.154-27.239, *P*＜0.001）；⑥淋巴结转移情况（有转移，HR=4.592，95%CI: 2.002-10.532，*P*＜0.001）；⑦病理类型是否相同（病理不相同，HR=0.127，95%CI: 0.055-0.293，*P*＜0.001）；⑧胸膜侵犯情况（HR=22.752, 95%CI: 7.730-66.965, *P*＜0.001）；⑨影像学特征（GGN-GGN组，HR=15.274，95%CI: 4.793-48.680，*P*＜0.001；GGN-GGO组，HR=5.394，95%CI: 1.660-17.529，*P*=0.005）。上述临床因子是影响同时性多原发肺癌的危险因子。

**2:** 临床病理特征与OS关系 The relationship between clinicopathologic characteristics and overall survival (OS)

Clinicopathologic characteristics	Number (%)	HR	95%CI	*P* value
Gender		5.090	2.105-12.310	＜0.001
Male	109 (30.53%)			
Female	248 (69.57%)			
Age		2.951	1.222-7.126	0.016
≤60 years	188 (52.66%)			
＞60 years	169 (47.34%)			
Smoking history		5.023	2.238-11.273	＜0.001
Yes	61 (17.09%）			
No	296 (82.91%)			
Sum of the diameters of the nodes		8.419	2.497-28.394	0.001
≤3 cm	317 (88.80%)			
＞3 cm	40 (11.20%)			
Diameter of the maximal node		9.269	3.154-27.239	＜0.001
≤2 cm	244 (68.35%)			
＞2 cm	113 (31.65%)			
Lymph node status		4.592	2.002-10.532	＜0.001
Positive	38 (10.64%)			
Negative	319 (89.36%)			
Location				
Same lobe	132 (36.98%)	1		
Ipsilateral but different lobe	128 (35.85%)	2.551	0.896-7.264	0.079
Bilateral	97 (27.17%)	1.549	0.491-4.888	0.455
Pathological pattern		0.127	0.055-0.293	＜0.001
Same	331 (92.72%)			
Different	26 (7.28%)			
Number of the lesions		0.225	0.049-1.025	0.054
2	269 (75.35%)			
≥3	88 (24.65%)			
Pleura status		22.752	7.730-66.965	＜0.001
Positive	65 (18.21%)			
Negative	292 (81.79%)			
Imageological characteristics				
GGN-GGN	34 (9.52%)	15.274	4.793-48.680	＜0.001
GGN-GGO	92 (25.77%)	5.394	1.660-17.529	0.005
GGO-GGO	231 (64.71%)	1		
Surgical method				
Lobectomy+lobectomy	138 (38.66%)	1		
Lobectomy+limited-resection	129 (36.13%)	1.253	0.464-3.385	0.656
Limited-resection+limited-resection	90 (25.21%)	2.079	0.760-5.690	0.154
OS: overall survival; HR: hazard ratio; CI: confidence interval; GGN: ground glass nodule; GGO: ground glass opacity.				

*Cox*多因素分析中有意义的临床因子分别为：①吸烟史（HR=4.771, 95%CI: 2.249-7.293, *P*=0.012）；②最大肿瘤长径（HR=6.737, 95%CI: 2.579-10.895, *P*=0.027）；③淋巴结转移情况（HR=3.753, 95%CI: 2.229-5.277, *P*=0.015）；④是否有胸膜侵犯（HR=9.876, 95%CI: 2.752-35.440, *P*＜0.001）。上述临床因子是影响sMPLC患者预后的独立危险因素。对上述独立危险因子做出各自的生存曲线见图 2。

结合表 2和图 1，可以看出，男性患者、有吸烟史、年龄大于60岁、各肿瘤长径之和大于3 cm、最大肿瘤长径大于2 cm、淋巴结有转移、胸膜受累、病理类型为非腺癌-腺癌组、肿瘤病理类型不相同、影像学表现为GGN-GGN或GGN-GGO的同时性多原发肺癌的预后较其它患者差。而各病灶位置（是否位于同一肺叶，是否位于同一侧肺）、肿瘤个数以及手术方式（*P*均＞0.05），并不影响患者的预后。

**1:**
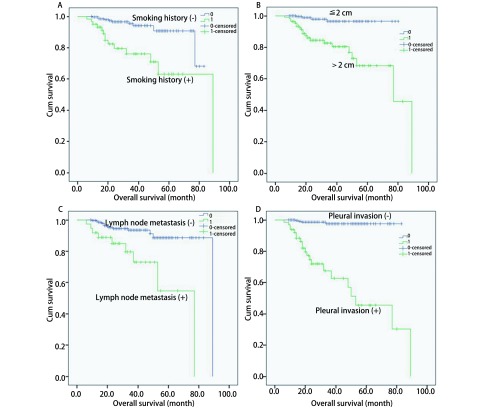
各个独立危险因子与总生存期的关系曲线。A：吸烟情况与术后总生存期的关系曲线；B：最大病灶长径与术后总生存期的关系曲线；C：淋巴结转移情况与术后总生存期的关系曲线；D：胸膜侵犯情况与术后总生存期的关系曲线。 Survival curves in sMPLC by the independent risk factors. A: Survival curve in sMPLC by smoking history; B: Survival curve in sMPLC by diameter of the maximal lesion; C: Survival curve in sMPLC by lymph node status; D: Survival curve in sMPLC by pleura status.

## 3 讨论

多原发肺癌，最初由Beyreuther^[6]^在1924年提出，以往认为sMPLC是一种较少见的疾病，但据近些年国内外的研究报道，其发病率呈不断上升趋势^[2, 3, 7-9]^。因而sMPLC成为胸外科临床医师必须面对的问题，多原发肺癌的诊断和治疗，特别是同时性多原发肺癌的诊断和治疗，尚有一定难度，因此，对该类疾病的研究具有重要的临床价值。

关于sMPLC的诊断，目前尚无统一完善的金标准。临床多参考Martini等^[5]^提出了诊断多原发肺癌的临床标准，由于该标准简便、可操作性强，一直以来为临床和病理医师广泛接受和采用。进入21世纪后，随着基因与分子生物学技术的不断发展，Martini标准也被不断补充和改进。美国胸科医师协会（American College of Chest Physicians, ACCP）先后3次对Martini-Melamed标准进行了补充^[10]^，其中补充的要点是在原来的基础上增加分子遗传学特征差异的鉴别。随后涌现了一些关于分子基因诊断的研究报道，如通过蛋白分子差异趋势^[11, 12]^、p53和*EGFR*突变^[13]^、二代测序^[14]^、比较基因组杂交技术（comparative genomic hybridization, CGH）等^[1]^对sMPLC的诊断有重要意义，但由于经济条件和可行性的制约，目前难以广泛应用于临床。sMPLC需要多学科综合诊断，诊断时需要综合考虑组织学类型、遗传学特点、影像学特征以及临床表现等加以诊断。

随着时间的发展，sMPLC的临床特征也在发生变化，以往报道的sMPLC病例多是多发的、较大的实性肿块。本研究总结显示，357例sMPLC的833个病灶中，表现为GGO的占79.23%（660/833），小于2 cm的病灶占83.19%。病灶位于右肺上叶者占35.77%；病例类型以腺癌为主（95.56%, 796/833），其次为鳞癌（2.40%, 20/833），与周丽娜等^[15]^报道一致。在796个腺癌中，浸润性腺癌占55.53%，微浸润腺癌和原位腺癌分别占23.99%和20.48%。肺腺癌亚型中，以腺泡样为主，占70.81%（313/442），其次是以乳头型为主的腺癌亚型，占26.24%，与李营等^[16]^报道的腺癌亚型以乳头型为主不同。sMPLC患者中女性明显多于男性，男女比0.44:1，与以往报道^[3, 5]^的MPLC好发于男性、以鳞癌为主不同，这可能与近年来肺癌治病因素的改变，使女性和腺癌的发病率升高。sMPLC常表现为多发GGO，临床分期较早，Ib期及以下分期占83.19%，其中原位腺癌占20.48%。本研究中，病理类型相同者发病率（92.72%）远高于不同病理类型（7.28%），其中以腺癌-腺癌为主（99.40%），其余2例为鳞癌-鳞癌。病灶位于双侧肺者97例（27.17%），同侧但不同肺叶者128例（35.85%），位于同一肺叶者132例（36.98%），与Yu等^[17]^的报道类似。

由于病灶大于2 cm的sMPLC预后明显比小于2 cm者差，所以建议行肺叶切除术+系统性淋巴结清扫术，对于较小的病灶行局部切除术，必要时术后辅以化学治疗或靶向治疗。对于病灶均小于2 cm的sMPLC的患者，如果病灶位置合适，尽可能地行局部切除术，特别是对于病灶为3个及3个以上的患者，应尽可能地保留健康肺组织及肺功能。对于病灶均位于同一肺叶的sMPLC患者，可以考虑行肺叶切除术。对于病灶位于双侧肺的sMPLC患者，多采用分期手术，两次手术时间间隔40天为宜，可以降低手术风险。遵循的手术原则是：先切除中央型、进展较快、病灶较大或伴有纵隔、肺门淋巴结转移的病灶，后切除周围型、进展较慢、病灶较小或无淋巴结转移的病灶，即先切除对预后影响较大、分期较晚的病变。此外，对于sMPLC患者，建议对病理标本做基因突变检测，为术后辅以靶向治疗提供应用指证。

本研究中位随访时间47个月，3年生存率91.93%，5年生存率84.37%，高于Takamochi等^[18]^报道的82.1%和77.3%与Yang等^[19]^报道的84.5%和75%，可能是近年多数sMPLC在早期被筛查出来并接受治疗的原因，使患者预后生存有所改善。本研究提示，位于双侧肺的多原发肺癌与位于同侧肺的多原发肺癌，预后无显著差异（*P*=0.151），位于同一肺叶的sMPLC与位于不同肺叶者的5年生存率亦无明显差异（*P*=0.793）。而日本的研究^[2]^提示病灶位于双侧者预后不良，这与病灶位于双侧者临床分期较晚有关，病灶位于同侧者准入相对谨慎，临床分期总体相对较早。对于sMPLC患者的手术方式，本研究表明叶切-叶切、叶切-局切（楔切或段切）和局切-局切3组预后生存无明显差异，与Trousse研究报道^[20]^的肺叶切除预后优于局部切除不同，可能是近些年来多数sMPLC在早期就被筛查出来并接受治疗的原因。有报道^[3]^称，全肺切除是患者预后的独立不良因素，应尽量避免，以保存足够的肺功能。此外，本研究还表明，虽然女性患sMPLC的风险高于男性，但男性sMPLC患者的预后却比女性差（HR=5.090, 95%CI: 2.105-12.310, *P*＜0.001），原因不明，或与男性吸烟有关。

本研究单因素生存分析提示，男性、有吸烟史、大于60岁、各病灶长径之和大于3 cm、最大病灶大于2 cm、有淋巴结转移、胸膜受累、不同病理类型、影像学表现为实性结节者较其他组预后差，与以往报道^[16, 21]^类似。多因素分析显示，只有有吸烟史、最大病灶大于2 cm、有淋巴结转移和胸膜受累为影响sMPLC预后生存的独立危险因素。此外，病灶均表现为GGO的患者预后明显较GGN组好，与以往的报道^[22]^相符。本研究结果显示，病灶个数并不是影响sMPLC预后的危险因素，sMPLC几乎与单原发肺癌有着相近的术后生存率。因此，sMPLC患者应该积极地接受手术治疗，而不是多发病灶认为是转移，贻误手术时机。

长期术后随访资料显示，同时性多原发肺癌患者的预后比肺内转移癌患者的预后好。因此，目前大多数学者主张，积极的外科手术是治疗同时性多原发肺癌最有效的方法。文献^[2, 17, 23, 24]^报道也显示，行手术治疗的sMPLC患者的生存期明显优于未行手术治疗的患者。在sMPLC的治疗方面，应把握以下几个原则：①在无手术禁忌证的情况下尽可能手术治疗；②尽可能完整、有效地切除肿瘤；③切缘宽裕的前提下，尽可能多地保留健康肺组织；④部分患者术后应采取多学科综合治疗（如化疗、靶向治疗等），以提高生存率。
